# Supplementary material for: RAS Subcellular Localization Inversely Regulates Thyroid Tumor Growth and Dissemination
Source: Cancers (Basel). 2020 Sep 10;12(9):2588. doi: 10.3390/cancers12092588 (PMC7565207; doi:10.3390/cancers12092588)
Supplement: Supplementary file 1 [file cancers-12-02588-s001.pdf]

Supplementary Materials: RAS Subcellular Localization Inversely Regulates Thyroid Tumor Growth and Dissemination

Yaiza García-Ibáñez, Garcilaso Riesco-Eizaguirre, Pilar Santisteban, Berta Casar and Piero Crespo

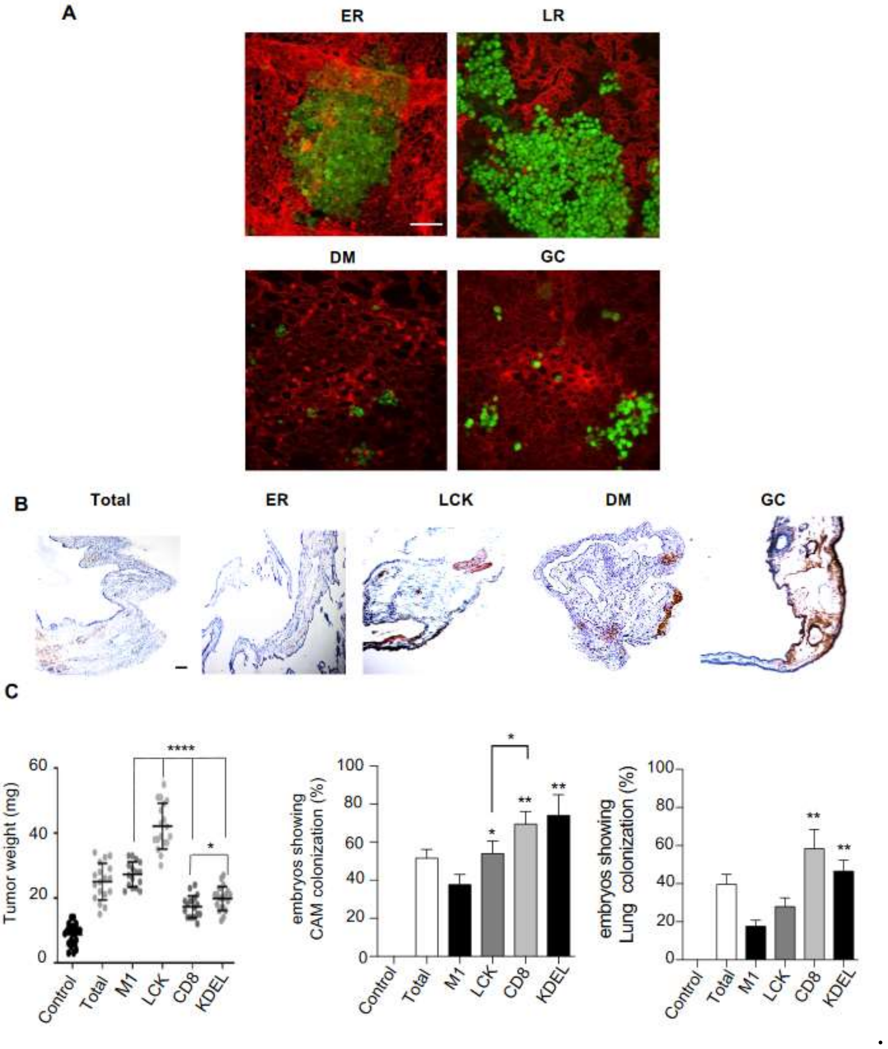

Figure S1. (**A**) Tumor formation by PCCL3 cells expressing HRAS at the indicated sublocalizations. Cells were pre-labeled with green fluorescence CellTracker and 20,000 cells were grafted on the CAM of live chick embryos grown ex ovo for 3 days. After 5 days, CAM vasculature was highlighted by injecting Rhodamine. Samples were visualized in a Leica Confocal Microscope using a 25× water objective. Images were reconstructed using Fiji Software. Scale bar = 200 µm. (**B**) Immunohistological analysis of colonized CAM. 106 cells were grafted onto the CAM of 10 days-old chick embryos. After 7 days distal CAM was collected. Rat cells were stained with an anti-RAT CD44 antibody (brown staining) (arrows) and tissues were counterstained with hematoxylin. Scale bars = µm. (**C**) NRAS site-specific effects on tumor growth and dissemination. 106 cells from each PCCL3-derived cell line stably expressing the indicated site-specific NRASV12 constructs were grafted on chick embryos and allowed to grow for seven days. (**A**) Tumor size. (**B**) Intravasation, expressed as the % of chicken displaying distal CAM colonization. (**C**) Distant metastases, expressed as the % of chicken displaying lung colonization. Data shows mean ± SD (weight); mean ± SEM (CAM and lungs) from three independent experiments using 9-15 embryos per case. * $p < 0.05$, ** $p < 0.01$ *** $p < 0.001$ and **** $p < 0.0001$ by double tailed unpaired Student T-test.

A

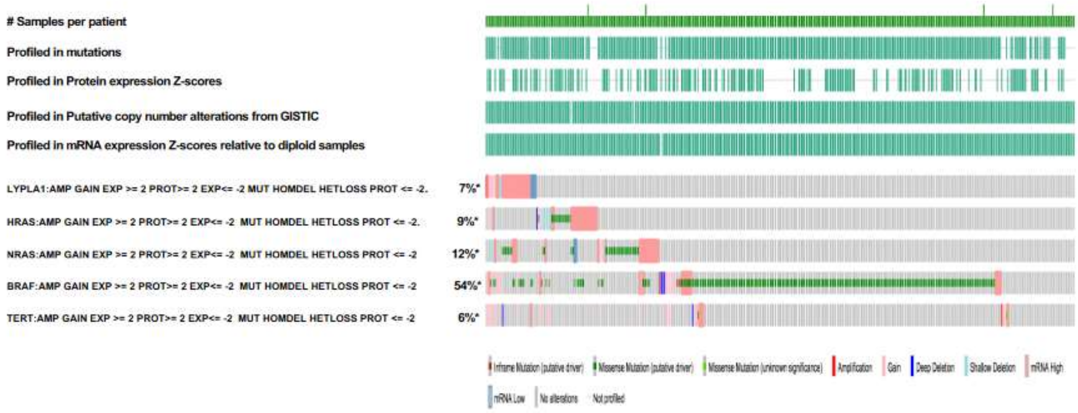

B

A	B	Neither	A Not B	B Not A	Both	Log2 Odds Ratio	p-Value	q-Value	Tendency
LYPLA1: AMP GAIN EXP>=2 PROT>=2 EXP<=-2 MUT HOMDEL HETLOSS PROT<=-2	TERT: AMP GAIN EXP>=2 PROT>=2 EXP<=-2 MUT HOMDEL HETLOSS PROT<=-2	184	12	8	6	>3	<0.001	**0.002**	Co-occurrence
LYPLA1: AMP GAIN EXP>=2 PROT>=2 EXP<=-2 MUT HOMDEL HETLOSS PROT<=-2	NRAS: AMP GAIN EXP>=2 PROT>=2 EXP<=-2 MUT HOMDEL HETLOSS PROT<=-2	169	11	23	7	2.225	0.006	**0.017**	Co-occurrence
LYPLA1: AMP GAIN EXP>=2 PROT>=2 EXP<=-2 MUT HOMDEL HETLOSS PROT<=-2	HRAS: AMP GAIN EXP>=2 PROT>=2 EXP<=-2 MUT HOMDEL HETLOSS PROT<=-2	176	14	15	5	2.067	0.023	**0.038**	Co-occurrence
LYPLA1: AMP GAIN EXP>=2 PROT>=2 EXP<=-2 MUT HOMDEL HETLOSS PROT<=-2	BRAF: AMP GAIN EXP>=2 PROT>=2 EXP<=-2 MUT HOMDEL HETLOSS PROT<=-2	70	7	122	11	-0.149	0.513	0.624	Mutual exclusivity

Figure S2. (**A**) Genetic alterations in APT 1(LYPLA1), HRAS, NRAS, BRAF and TERT in thyroid carcinomas. Obtained from a cohort of 507 patients, at cBioPortal for Cancer Genomics database (TCGA). (**B**) Mutual Exclusivity analysis for the same set of data in the Oncoprint.

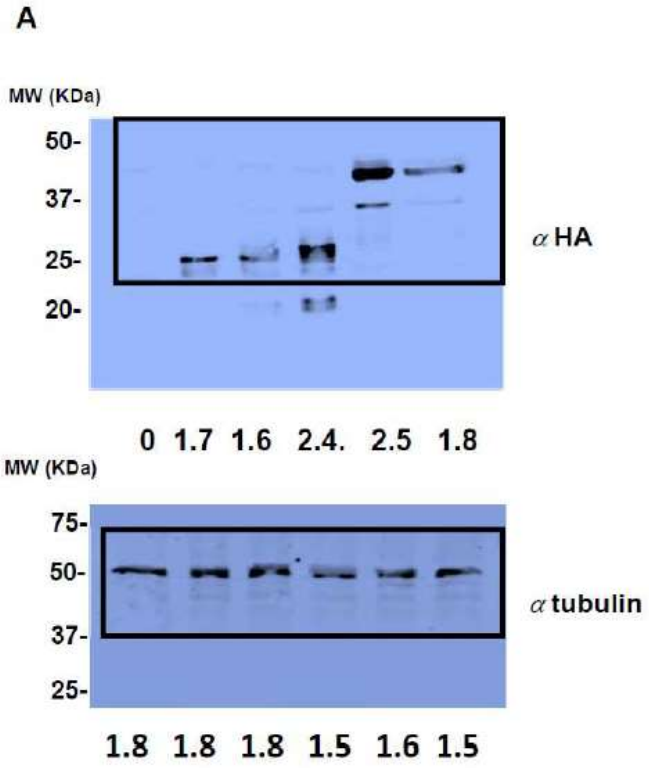

Figure S3. Unprocessed images for Western Blot results corresponding to the Figure 1A.

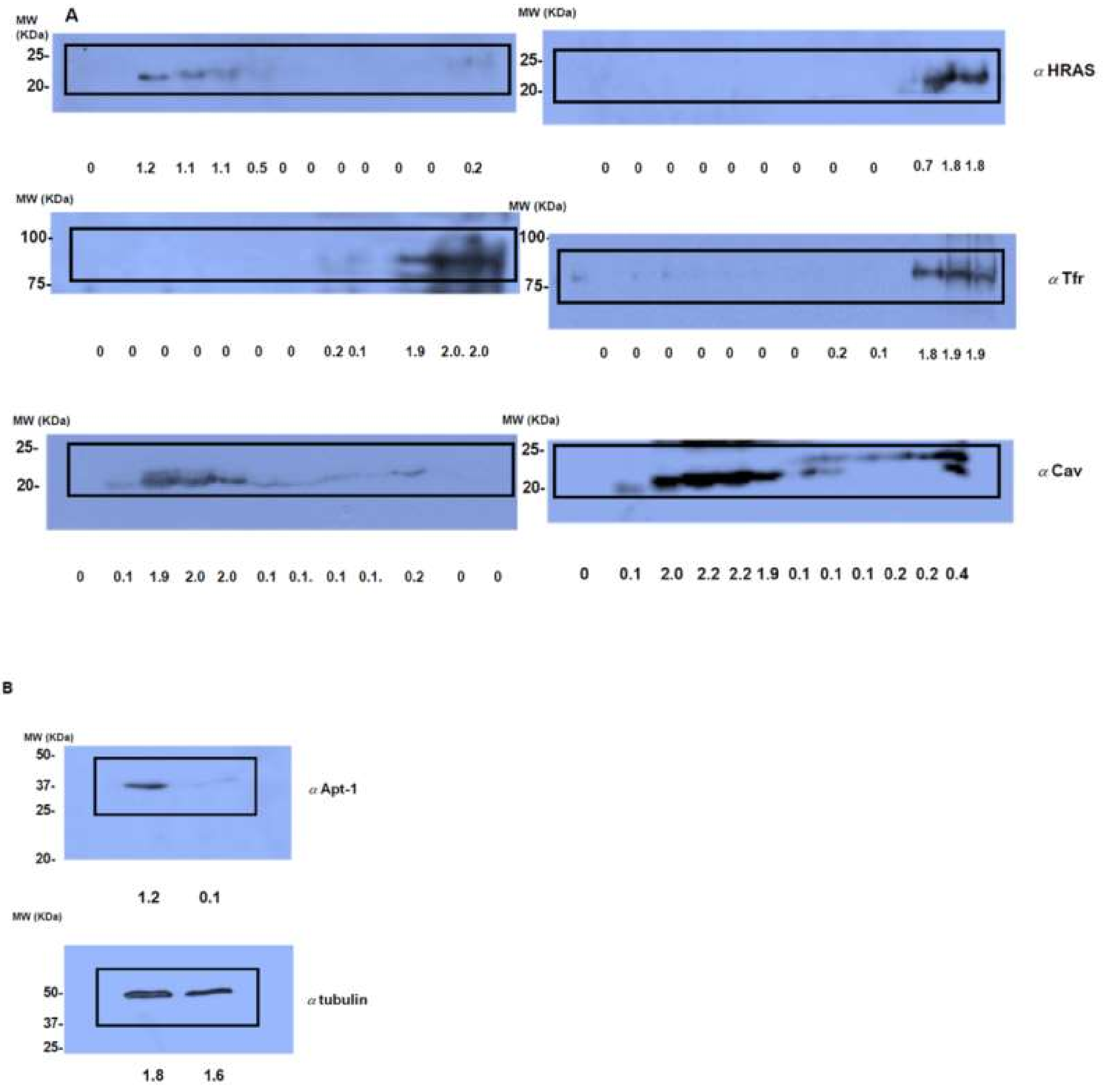

Figure S4. Unprocessed images for Western Blot results corresponding to the Figures 3A, 3B.

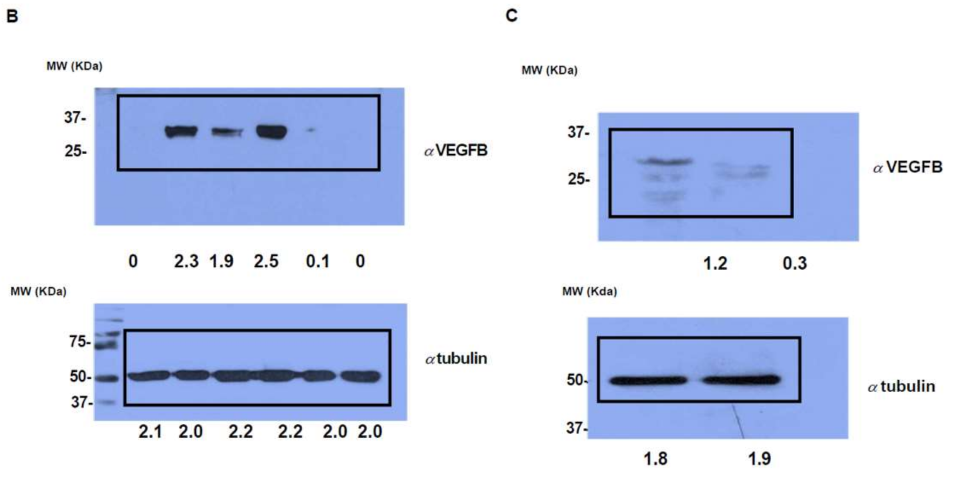

Figure S5. Unprocessed images for Western Blot results corresponding to the Figures 4B, 4C.

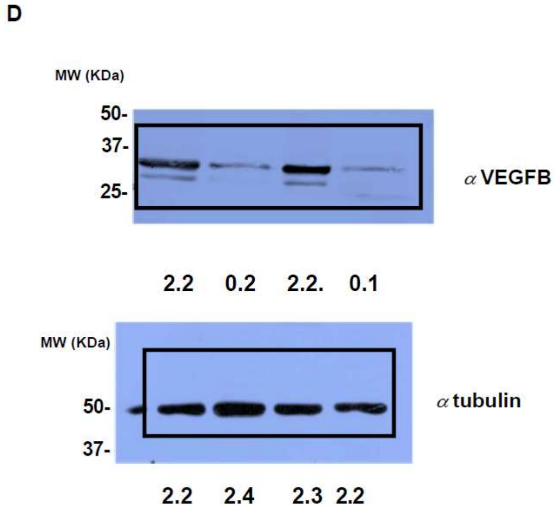

Figure 6. Unprocessed images for Western Blot results corresponding to the Figures 5D.

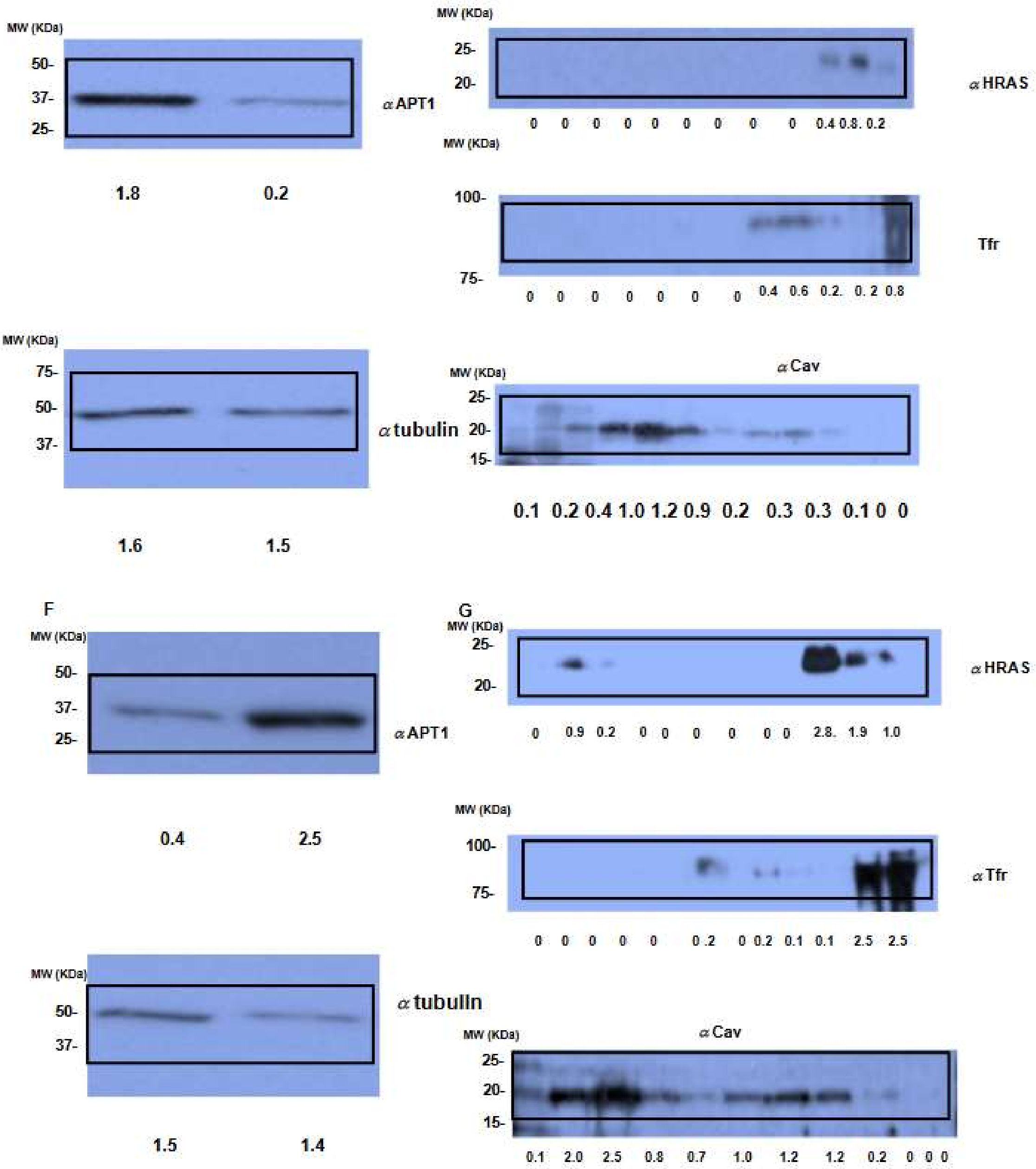

Figure S7 Unprocessed images for Western Blot results corresponding to the Figures 7A, 7B, 7F, 7G.

© 2020 by the authors. Licensee MDPI, Basel, Switzerland. This article is an open access article distributed under the terms and conditions of the Creative Commons Attribution (CC BY) license (http://creativecommons.org/licenses/by/4.0/).
